# Access, use and disposal of antimicrobials among humans and animals in Wakiso district, Uganda: a qualitative study

**DOI:** 10.1186/s40545-021-00361-4

**Published:** 2021-08-20

**Authors:** David Musoke, Carol Namata, Grace Biyinzika Lubega, Freddy Eric Kitutu, Lawrence Mugisha, Saba Amir, Claire Brandish, Joviah Gonza, Deborah Ikhile, Filimin Niyongabo, Bee Yean Ng, Jean O’Driscoll, Kate Russell-Hobbs, Jody Winter, Linda Gibson

**Affiliations:** 1 Department of Disease Control and Environmental Health, School of Public Health, College of Health Sciences, Makerere University, P. O. Box 7072, Kampala, Uganda; 2 Sustainable Pharmaceutical Systems (SPS) Unit, Department of Pharmacy, School of Health Sciences, College of Health Sciences, Makerere University, P. O. Box 7072, Kampala, Uganda; 3 College of Veterinary Medicine, Animal Resources and Biosecurity (COVAB), Makerere University, P. O. Box 7062, Kampala, Uganda; 4 School of Animal, Rural and Environmental Sciences, Nottingham Trent University, Nottingham, NG25 0QF UK; 5 Buckinghamshire Healthcare NHS Trust, Aylesbury, HP21 8AL UK; 6 Institute of Health and Allied Professions, School of Social Sciences, Nottingham Trent University, Nottingham, NG1 4FQ UK; 7 Department of Biosciences, School of Science and Technology, Nottingham Trent University, Nottingham, NG11 8NS UK

**Keywords:** Antimicrobial resistance, Antimicrobials, Antimicrobial stewardship, Community, Humans, Animals, One Health, Waste management, Uganda

## Abstract

**Background:**

Inappropriate use of antimicrobials in both humans and animals is a key driver of antimicrobial resistance (AMR). In addition, human behaviours such as poor disposal of antimicrobials in the environment can increase their exposure to microbes which can impact on humans and animals. However, evidence on access, use and disposal of antimicrobials for humans and animals at community level in Uganda is limited. This study therefore explored access, use and disposal of antimicrobials among humans and animals in Wakiso district, Uganda.

**Methods:**

A qualitative study was conducted that involved focus group discussions (FGDs) and key informant interviews (KIIs). Participants of the FGDs were community health workers (CHWs) and farmers involved in animal husbandry, while key informants included: officials from the Ministry of Health; Ministry of Agriculture, Animal Industry and Fisheries; human and animal health professionals; district health officials; and members of the national AMR surveillance committee. Twelve FGDs were held (8 for CHWs and 4 for farmers) while 15 KIIs were conducted. Thematic analysis in NVivo (version 12) was performed.

**Results:**

Five main themes emerged from the study: access to antimicrobials in humans; access to antimicrobials in animals; use of antimicrobials in humans; use of antimicrobials in animals; and disposal of antimicrobials. Community members mainly accessed antimicrobials for humans from public health facilities such as government health centres, as well as private facilities, including drug shops and clinics. Antimicrobials for animals were obtained from veterinary practitioners and drug shops (both for humans and veterinary). Examples of inappropriate use of antimicrobials in both humans and animals was evident, such as sharing antibiotics among household members, and giving human-prescribed antimicrobials to food-producing animals as growth promoters. While some CHWs returned unused antimicrobials to public health facilities for proper disposal, community members mainly disposed of antimicrobials with general household waste including dumping in rubbish pits.

**Conclusions:**

There is a need to increase awareness among the population on proper access, use and disposal of antimicrobials for both humans and animals. Development of a drug disposal system at community level would facilitate improved waste management of antimicrobials. Together, these measures would help prevent the rate of progression of AMR in communities.

## Introduction

Access to antimicrobials has generated impressive improvements in global health in recent years especially in low- and middle-income countries (LMICs). The significant reduction in morbidity and mortality from infectious diseases has been linked in part to increased access and use of antimicrobials [1–3]. However, inappropriate use and exposure of antimicrobials in humans, animals and the environment is thought to be one of the key drivers of antimicrobial resistance (AMR). Antibiotics are among the most misused medicines around the world due to their ubiquitous availability, affordability and perceived safety, which has contributed to the growing public health crisis of AMR [4]. Although the emergence of AMR is an inevitable consequence of antibiotic use, misuse and overuse of antibiotics accelerates the process of AMR development, limiting the useful lifespan of available drugs for the treatment of bacterial infections [5]. Globally, approximately 700,000 people die from antimicrobial-resistant infections every year, and AMR-related mortality is predicted to rise to 10 million per year by 2050 [6]. It has been reported that the majority of the world’s annual 5.7 million antimicrobial-treatable deaths occur in LMICs [7, 8]. In many LMICs including Uganda, infections such as gonorrhoea, tuberculosis, bacterial bloodstream infections, urinary tract infections, and foodborne diseases have become increasingly difficult to treat leading to increased risk of spreading resistant infections, higher treatment costs, and increased risk of death [4, 7, 9, 10]. Although AMR is a global challenge, its effects are likely to be felt most acutely in LMICs constrained with insufficient health infrastructure, challenges with infection prevention and control, insufficient access to alternative treatment options, as well as inadequate human and financial resources to counter drug-resistant infections [11].

Access to antimicrobials by households is largely dependent on geographical location in relation to health service providers and economic status. The Uganda Health Sector Development Plan 2015/16–2019/20 indicated that only 72% of households live within 5 km of a health facility [12]. Although this is indicative of a geographical improvement in access to health services compared to previous years, affordability of services remains a limiting factor. The primary sources of health care financing are individual households out-of-pocket expenditure (37%), donors (45%) and government (15%) [12, 13]. Despite the accessibility and financial challenges, there are reports of high and unnecessary use of antimicrobials among patients. Examples of such practices include use of antimalarials for fevers not associated with malaria, as well as use of antibiotics for non-bloody diarrhoea and non-pneumonia upper respiratory infections that are commonly caused by viruses [14]. Uganda has also reported resistance against commonly used antimicrobials including ampicillin, ciprofloxacin and ceftriaxone used for bacterial infections, artemether–lumefantrine commonly used for malaria, as well as rifampicin and isoniazid used for tuberculosis [10, 15]. Consequently, second- or third-line antimicrobials, which are more expensive and not readily available in health facilities, might be required to treat these common infections [12, 16].

Antimicrobials in Uganda are categorized under Class B by the National Drug Authority (NDA)—the regulatory body for medicines in Uganda—hence these require a prescription before being dispensed. However, in practice, many of them are easily attainable from drug outlets such as pharmacies and drug shops without a prescription [10]. Self-medication and premature discontinuation of antimicrobials has also been observed in Uganda contributing to the development of AMR [9, 10]. These undesirable practices are drivers of the growing drug resistance of microorganisms across the country. In the animal industry, misuse and consumption of antimicrobials is rampant and estimated to be higher than in human health [17]. This is because over the past 60 years, antibiotics have been used in animal feed at sub-therapeutic doses to improve animal performance and for feed efficiency, in addition to their use for treating infections [18]. The need for in-feed antibiotics as growth promoters has been driven by the demand for animal protein sources due to the rapidly increasing human population leading to higher antimicrobial usage in animal farming [19]. Without the use of antimicrobials, especially antibiotics, production costs would be higher with lower yield [20]. However, antibiotic use in animals is poorly documented for many LMICs, and this is exacerbated by weak enforcement of policies on antimicrobial use [15]. In Uganda, farmers often self-prescribe antibiotics for their animals when they fall ill and purchase them from veterinary drug shops without the directives of veterinarians or a prescription [10]. In addition, it has been reported that some farmers administer human-prescribed antiretroviral drugs to livestock as ‘immune boosters’ [21]. Such use of antimicrobials in food animals carries additional risks to human health because resistant microbes and antimicrobial residues have been found in both living and ready-to-eat animal products [19, 22]. In addition, antimicrobial residues are extensively found in the environment including soil and water [20]. Environmental exposure of microbes to antimicrobials is aggravated by their poor disposal particularly in LMICs [23]. Inappropriate disposal of antimicrobials such as dumping in the environment together with other household waste has also been documented in several studies [24–26].

Antimicrobials and their residues can be found almost everywhere in the environment, and antimicrobial-resistant organisms continue to interact between humans, animals and the environment [20]. This emphasizes the need for a One Health approach when tackling AMR globally but especially in LMICs including Uganda. The Government of Uganda has increased attention to promoting Antimicrobial Stewardship (AMS) in recent years including development of an AMR National Action Plan (NAP) [9]. The NAP emphasizes the need to strengthen the evidence base on antimicrobial use to inform policy including through research [9]. However, most of the research on AMS in the country to date has been from large health facilities [27–29], with minimal literature from community settings. Given that AMR is a complex public health problem that concerns many sectors, there is need for more evidence on access and use of antimicrobials among humans and animals particularly in the community, as well as disposal of antimicrobials at household level. Such evidence is key to inform medical, veterinary, environmental and other professionals as well as policy makers on preventing the further development of AMR through a One Health approach. The use of qualitative methods is particularly important to critically examine behaviours and practices of the community regarding antimicrobials which may not be achieved quantitatively. In addition, qualitative research enables deeper exploration of concerns and understanding of myths regarding antimicrobials established by other studies. Therefore, this study qualitatively explored access, use and disposal of antimicrobials among humans and animals in Wakiso district, Uganda.

## Methods

### Study design and participants

The qualitative study conducted in Wakiso district used focus group discussions (FGDs), and Key Informant Interviews (KIIs) to explore access, use and disposal of antimicrobials among humans and animals in the community. Participants of the FGDs were community health workers (CHWs) and farmers involved in animal husbandry who were purposively selected in consultation with village leaders and community mobilisers in the study area. CHWs were involved in the study as they are the first point of contact of the community with the health system and are normally knowledgeable about health-related issues in their locality. In addition, many CHWs are involved in providing treatment for malaria, pneumonia and diarrhoea in their villages among children under 5 years of age under integrated community case management of childhood illnesses. Some CHWs own animals therefore they can also share their experiences on use of antimicrobials in animals in their households. Farmers involved in animal husbandry were included in the study to provide their experiences on access and use of antimicrobials for their animals, as well as for themselves and other household members. Twelve mixed-gender FGDs, each comprising 8 participants, were conducted, with 8 among CHWs and 4 among farmers. A total of 15 key informants were purposively selected based on their expertise and relevance to AMR and AMS for both human and animal health at national, sub-national and local levels. These included: health professionals from public and private health facilities; veterinary practitioners; members of the Wakiso district health office; Ministry of Health officials from the pharmacy and health promotion departments; Ministry of Agriculture, Animal Industry and Fisheries officials; and members of the national AMR surveillance committee. The 12 FGDs and 15 KIIs were sufficient to reach data saturation.

### Study area and setting

The study was carried out in Kajjansi and Kasanje town councils in Wakiso district, Uganda, for the FGDs. However, the KIIs involved national-level stakeholders beyond the district. These town councils were purposively selected for their involvement in the study as they have a good representation of peri-urban and rural communities, and are highly involved in animal husbandry. Wakiso is the most populated district in Uganda with a population of 2,007,700 as per the 2014 national census and an estimated annual growth rate of 4.1% [13]. The district consists of four municipalities, seven sub-counties and eight town councils. The district includes both urban and rural areas, with half of its population living in urban settings [13]. Wakiso district has 589 health facilities, comprising 72 government, 477 private-for-profit, and 40 private not-for-profit facilities [30]. Kajjansi town council has 3 government health facilities (one health centre II, one health centre III, and one health centre IV) while Kasanje town council has only one government health facility (health centre III). Both town councils have several private health facilities including health centres, clinics, pharmacies and drug shops which are all involved in health service delivery including dispensing antimicrobials. The district has government veterinarians who are mandated to oversee and support farmers in the community. In addition, Wakiso district has several private veterinarians who are involved in managing illness in animals. Wakiso’s proximity to Kampala, the country’s capital city, provides easy access to antimicrobials that may be unavailable or more expensive within the district.

### Data collection

FGD and KII guides were used to collect data from the respective participants. These tools were developed following a comprehensive literature review on antimicrobial access, use and disposal in Uganda and other LMICs. The tools were piloted in a village in Kajjansi that was not involved in the actual study. Specifically, the tools assessed for both humans and animals: sources of antimicrobials (private and public sectors, markets, veterinary officers, and drug shops/pharmacies); ease of access to antimicrobials; self-prescription; free versus paid for antimicrobials; duration of consumption of antimicrobials; sharing of antimicrobials between household members and different animals; use of left-over antimicrobials; and disposal of expired/unused antimicrobials. The guides were developed in English, and later translated to *Luganda*, the local language most used in the study area. All FGDs were conducted in the local language and facilitated by two members of the research team who were experienced in qualitative research. One of the members moderated the discussions while the other helped with notetaking as well as audio recording of the proceedings. The KIIs were conducted by one of the researchers who audio recorded them. Most of the KIIs were conducted in English, except for a few held in the local language*.* For Kajjansi, the FGDs were held at a project field office in the area, while in Kasanje, the home of a village chairperson was used. KIIs were scheduled by telephone appointment to ensure that the participants were available to take part. The key informants suggested the appropriate date, time and location for the interviews which were predominantly held at their workplaces including health facilities and offices. The FGDs and KIIs were conducted concurrently. While the FGDs lasted between 30 and 60 min, the KIIs took between 20 and 40 min.

### Data management and analysis

All audio recordings of the FGDs and KIIs were transcribed verbatim in the appropriate language (English or *Luganda*) by one of the researchers involved in data collection. Two researchers read the transcriptions to ensure they were a true representation of the data collected. Once the transcripts were validated, those in *Luganda* were translated to English by one of the researchers. All transcribed data were then transferred to NVivo (version 12) for analysis. Data from the FGDs and KIIs were analysed together since the two data collection methods were used for the same research question. However, data from each of the methods was clearly identifiable during data analysis. Indeed, the results presented in the manuscript highlight which findings came from the FGDs or KIIs. During data analysis, the imported texts were read which helped the researchers to further appreciate the data, which was then analysed by data coding. Data coding involved identifying and extracting passages of text from the transcripts into nodes. These nodes were created in NVivo to represent each text of the transcript as a code. To code a chunk of data under a node in each transcript, the text was highlighted and pulled to the identified free node. In some cases, multiple nodes were assigned to the same chunk of text. After coding the text, a search was conducted to find connections between the various nodes. Similar nodes were identified and grouped together conceptually to form tree nodes, and each tree node was designated as a sub-theme. Using thematic analysis, the tree nodes were then grouped together into 5 main themes. These themes are employed to present the major findings of the study.

### Ethical considerations

Ethical approval for the study was obtained from Makerere University School of Health Sciences Research and Ethics Committee (2019-051). The study was also approved and registered by the Uganda National Council for Science and Technology (HS 2711). All participants provided written informed consent before taking part in the study. Data were only accessed by the research team and used solely for purposes of the study.

## Results

The results of this study are presented under 5 main themes that emerged from the FGDs and KIIs: access to antimicrobials in humans; access to antimicrobials in animals; use of antimicrobials in humans; use of antimicrobials in animals; and disposal of antimicrobials. The study established that community members accessed antimicrobials for humans mainly from public health facilities such as government health centres, as well as private facilities including drug shops and clinics. Antimicrobials for animals were obtained from veterinary practitioners and drug shops (both for humans and veterinary). The study found inappropriate use of antimicrobials for both humans and animals, such as sharing antibiotics among household members, and giving human-prescribed antimicrobials to food-producing animals as growth promoters. In addition, it was established that while there was disposal of unused antimicrobials at public health facilities by CHWs, community members mainly disposed of antimicrobials as part of general household waste including dumping in rubbish pits.

### Access to antimicrobials in humans

#### Public health facilities

CHWs and farmers reported that they accessed antimicrobials from public health facilities such as government health centres. It was revealed that these centres prescribed antimicrobials to patients after making a clinical assessment and diagnosis. In addition, it was reported that antimicrobials for sick children aged below 5 years were also accessed from CHWs who are involved in treatment of childhood illnesses of malaria, pneumonia and diarrhoea.

*“Most of the time, we go to Nakawuka government health centre where they give us medicine after conducting a medical check-up. That is why we go to Nakawuka because of the assurance that they will write a prescription after carrying out some medical tests which is not always the case elsewhere.”* CHW, participant 3, FGD 11

Although most CHWs and farmers reported accessing antimicrobials from government health facilities, they faced various challenges whilst doing so, including travelling long distances. Despite the government health facilities providing antimicrobials free of charge, many community members found the high transport costs incurred to get to the facilities prohibitive. As such, some individuals resorted to obtaining antimicrobials from alternative sources.

*“Distance is a big challenge because I stay in Buswa and the government health centre is in Mpumudde so it makes it hard for me to go there for health care. If I ask a boda boda* [commercial motorcycle] *rider to take me to the health centre and back, they may ask for three thousand shillings* [approximately USD 0.8] *which I may not have. Therefore, if I have no money for transport, I give up and resort to other options.”* CHW, participant 1, FGD 9

Another challenge faced by community members established from the FGDs were the long waiting times reported at government health facilities before being attended to by health professionals. Participants reported that they sometimes spent a lot of time waiting to see health workers only to be referred to alternative places that had the recommended drugs due to regular stockouts at the facilities. This led to participant perceptions that public health facilities could not guarantee the availability of necessary medicines. Therefore, instead of waiting at the public facilities, some participants said that they visited alternative places where they quickly got the required service hence saving time for work and other activities.

*“Truth be told, I do not go to public health centres for medication. I cannot waste my time standing in a long queue just to get paracetamol and then be told to go buy the rest of the medication from a pharmacy. I am a parent and have to meet the needs of my family such as school fees. So, I would rather get some little money and go to a pharmacy or drug shop to buy drugs in the shortest time possible so I can spare time to attend to my family needs.”* Farmer, participant 5, FGD 6

#### Private health facilities

From the FGDs, community members accessed antimicrobials from private health facilities such as clinics, pharmacies and drug shops. They accessed these facilities mainly because many were located near their homes, and there was more confidence that they would have stock of antimicrobials, perceived to be frequently unavailable at government health facilities. It was established that private health facilities also offered shorter waiting times to get a service compared with government facilities.

*“When community members visit the government health facility and find no drugs, the next time they fall sick they do not return to the facility as they still think that it’s not worth it as they may still find no drugs. Therefore, they often ignore the option of visiting the health facility again for medical help and instead go to private clinics and drug shops.”* CHW, participant 2, FGD 1

From the experience of CHWs and farmers who accessed government health facilities where antimicrobials were unavailable, they were always given prescriptions to enable them to purchase the required medicines from private health facilities. Therefore, the unavailability and stock-out of certain antimicrobials at government health facilities promoted purchase of drugs from private providers.

*“In some communities, it is very hard to access medicines from government health centres as they are rarely available there so most people resort to drug shops or pharmacies. The health workers at the health centre write prescriptions for us to get the unavailable medicines from private pharmacies.”* Farmer, participant 3, FGD 4

Although the FGDs established that community members routinely accessed antimicrobials from private health facilities, they were faced with the challenge of high cost. Indeed, many participants reported that they could not always afford to purchase a complete course of antimicrobials from private health facilities. As such, they sometimes purchased inadequate doses depending on their financial ability, and as soon as the disease symptoms subsided, some never returned to purchase the remaining doses. In addition, it was established that many private providers such as drug shops issued antimicrobials to community members without any prescription or medical investigation. This situation was reportedly further exacerbated by the need to make profit in the private sector.

*“We strive to get the complete dosage from the drug shops. But in case there is not enough money, we first buy what we can afford, and then buy the rest when we get more money. This means that in case the money is not obtained at the appropriate time, one will not get the whole dosage as it is supposed to be.”* CHW, participant 8, FGD 8

### Access to antimicrobials for animals

#### Veterinary practitioners

Most farmers reported that they accessed antimicrobials for their animals from private veterinary practitioners. It was revealed that these were mainly mobile veterinary practitioners who travelled from village to village treating animals. However, some of these practitioners lacked adequate diagnostic equipment for the animals which increased the risk of misdiagnosis. Farmers also reported that private veterinary practitioners were expensive which was a major hindrance to using their services. However, since the public veterinary practitioners as part of local government were inactive, community members had no choice but to opt for the expensive private veterinary workers.

*“In my community, there are government public veterinary practitioners. However, the ones who were assigned to our village are very inactive as they hardly ever check on our sick animals. Therefore, when our animals fall sick, we have to call a private veterinary practitioner who is very expensive.”* Farmer, participant 1, FGD 10

#### Veterinary drug shops

Farmers reported that they accessed antimicrobials for their animals from veterinary drug shops often with recommendations from veterinary practitioners. However, it was established that there were few veterinary drug shops in their communities. As such, farmers faced a challenge of travelling long distances to access some of these drug shops. It was also noted that at times antimicrobials purchased from these facilities were found to be expired hence affecting their effectiveness in treating animal infections.

*“For animal antimicrobials, we have to travel long distances for example to Mpigi to find veterinary drug shops where they are sold. However, sometimes the drugs are expired and cannot be effective in treating the animal diseases even after travelling such a long distance to access them.”* Farmer, participant 2, FGD 4

Some farmers revealed that they used previous prescriptions from veterinary practitioners to purchase antimicrobials from veterinary drug shops. This was partly because the farmers believed that they had gained enough experience in managing their animals for many years. Therefore, they did not feel it necessary to seek advice from veterinary practitioners anymore. However, it was reported by the key informants that farmers who purchased antimicrobials without consulting veterinary practitioners often gave the wrong doses to their animals. This was evidenced when the veterinary professionals interacted with farmers during their visits to the community.

“*We used to buy antimicrobials from veterinary drug shops in our village. Although over the years, we got to know the common drugs used to treat our animals. Therefore, we don’t call veterinary practitioners anymore but use previous prescriptions to buy drugs for our sick animals.*” Farmer, participant 8, FGD 6

#### Human drug shops

From the FGDs, some participants reported that they accessed antimicrobials for their animals from drug shops selling medicines primarily for humans. Farmers revealed that they purchased and used antimicrobials prescribed for humans after receiving testimonies from fellow farmers that they were effective in treating animal infections. Additionally, farmers reported that they sometimes accessed antimicrobials for their animals from human drug shops due to proximity to their homes in comparison to veterinary drug shops. As such, they did not need to travel long distances, costing the farmers less in terms of travel costs and time, to access antimicrobials for use among their animals.

*“The animal drug shops are not readily accessible, and it is now common knowledge that medicines given to humans can be used to treat animals. So instead of moving a long distance to the animal drug shops, we buy antimicrobials that we use when we are sick from human drug shops which have proved to yield positive results in treating animal diseases.”* Farmer, participant 2, FGD 6

### Use of antimicrobials in humans

#### Inappropriate use of antimicrobials by community members

The study established that most community members used antimicrobials inappropriately. For instance, some FGD participants reported that they shared antimicrobials with other family members in case more than one person in the household was sick. In addition, the sharing of antimicrobials was reported as a common practice among children of the same household whose parents or guardians could not afford to purchase complete courses for each sick member of the family.

*“I may go to the drug shop and buy medicine for my sick child and on reaching home, I find that another child is sick. I therefore immediately share the medicine among the sick children because there is nothing else I can do at that moment with no funds to buy another dose.”* Farmer, participant 6, FGD 10

Another improper practice by community members was self-medication. Instead of visiting health professionals for diagnosis and prescription, it was established from the KIIs that many community members self-prescribed antimicrobials to treat self-diagnosed symptoms and conditions. It was therefore believed that as a result, patients often consumed unnecessary or inappropriate courses of antimicrobials. It was further established that some community members who self-medicated used previous prescriptions to purchase antimicrobials without recommendation from a health worker.

*“Self-medication is very common because a patient can come here and I prescribe for them antimicrobials, and the next time that person is sick, they will not come. They will use the same labelled packet if they have the same symptoms to purchase the same drug from a private health facility.”* Health practitioner, Kajjansi Health Centre IV

From the KIIs, self-medication by community members was also noted to result in drug overuse. As an example, it was established that there was misuse of antibiotics such as amoxicillin by community members.

*“For some people, you find that even if they do not need antibiotics, they still access and use them. They keep saying, ‘For me, if I do not swallow that medicine* [amoxicillin]*, I will not get better’. So, they make it a habit to take such antimicrobials even when not necessary.”* Health practitioner, Kasanje Health Centre III

Consumption of incomplete courses of antimicrobials was also practised by community members. It was established from the FGDs and KIIs that some community members discontinued the course of antimicrobials in cases where their symptoms subsided. Whereas some community participants reported disposing of the left-over antimicrobials, others kept them for future use in case of recurring infection or if another family member presented with the same symptoms. Some FGD participants reported discontinuation of the course of antimicrobials due to busy work schedules. It also emerged that some community members discontinued medication because of side effects.

*“I take the medication as prescribed, but as soon as I feel better, I keep the rest for future use when I become sick again or when another family member becomes sick. This is a common practice within our community.”* Farmer, participant 6, FGD 6

### Use of antimicrobials in animals

#### Improper use of antimicrobials among animals

It was established that some farmers offered incomplete courses of antimicrobials to their animals. This was because when the symptoms of their sick animals decreased, farmers withdrew medication and kept it for use in case of future infections. However, it was reported by the key informants that some farmers tended to unintentionally overdose their animals due to not following instructions from veterinary practitioners. This was evidenced when the veterinary workers interacted with the farmers on various occasions. As such, overdosage was believed to contribute to accumulation of antimicrobial residues in animal food products such as meat which was later sold for human consumption.

*“Another problem we find is that some farmers overdose the animals unknowingly hence you find that the meat has high amounts of drug residues, yet they continue to sell or consume such products themselves which is not a good practice.”* Veterinary practitioner, Kajjansi town council

### Use of human antimicrobials in food-producing animals

It was established from the KIIs that some farmers used antimicrobials prescribed for humans in food-producing animals for non-pharmaceutical purposes. Key informants reported that human antimicrobials particularly antibiotics were used to preserve animal products such as meat meant for human consumption. It was also established that some community members added human-prescribed antimicrobials, such as antiretroviral drugs to animal feeds for growth promotion especially for pigs.

*“There are people who actually use human prescribed drugs in animals. You may find somebody who is HIV positive and thinks the antiretrovirals are also good for their animals so they go ahead and give some of the drugs to pigs to enhance their growth.”* Veterinary practitioner 1, Wakiso district

### Disposal of antimicrobials

#### Household disposal

Regarding disposal of antimicrobials, it was reported from the FGDs that community members disposed of left-over, unused, unwanted and expired antimicrobials together with general waste for example in rubbish pits located in their homes. The study also established that such antimicrobials were sometimes burned or dumped in the bush together with other household waste. Some participants explained that disposal of antimicrobials in rubbish pits or the bush had the potential for them being accessed by children, which could lead to adverse health effects if ingested. As such, participants suggested changes to how antimicrobials were disposed of including using pit latrines or burying them to ensure they were out of children’s reach.

*“In case of spoiled or expired drugs, it is better to dig up a hole in the ground and bury them or throw in a pit latrine rather than throwing them around the compound or the bush which could predispose children to the risk of intoxication.”* Farmer, participant 6, FGD 4

#### Disposal at public health facilities

Some CHWs reported that they took unwanted or expired antimicrobials to government health facilities for final disposal. However, disposal at government facilities was limited to CHWs who were involved in treatment of childhood illnesses. These CHWs reported that they received safety boxes from government health facilities. These boxes were used to store expired, left-over or spoilt antimicrobials, and other medical waste such as used malaria Rapid Diagnostic Test (RDT) kits. When these safety boxes became full, they were returned to the nearby government health facility for safe disposal of the waste.

*“Some of us were given safety boxes where we keep the spoilt medicines. When the box gets full, one takes the responsibility to return it to the government health centre for disposal. The medicines we dispose in the boxes include both personal ones and those we use for treatment of sick children in the community.”* CHW, participant 2, FGD 12

## Discussion

Global improvements in the use of antimicrobials have resulted in substantial gains in health care delivery [2]. Increased access to antimicrobials remains a crucial component in the management of infectious diseases. However, misuse of antimicrobials in both humans and animals has contributed to the global challenge of AMR which remains a grave public health threat. This study, which explored access, use and disposal of antimicrobials among humans and animals in Wakiso district, Uganda, showed that while antimicrobials for human use are free at public facilities, community members routinely used private facilities due to health system challenges plaguing the public centres. Regarding access to antimicrobials for use in animals, the study established that these were predominantly obtained from private facilities, including veterinary practitioners, as well as veterinary and human drug shops. Consistent with existing evidence [31, 32], our study further demonstrated inappropriate use and disposal of antimicrobials both in humans and animals in the community. These findings provide evidence on antimicrobial use as recommended by the Uganda AMR NAP and demonstrate the need for a One Health approach in ensuring effective access and use of antimicrobials in both humans and animals. This recognition of the need for a One Health approach in addressing the misuse of antimicrobials in the fight against AMR has been previously articulated by Musoke et al. [33] and Pokharel et al. [34]. In addition, our findings emphasize the importance of implementing the World Health Organization (WHO) global recommendations for containment of AMR such as improving use of antimicrobials, reducing the use of antimicrobials in food-animal production, and improving access to appropriate antimicrobials [35].

Our study revealed that access to antimicrobials is partly provided through the public primary health care system, including government health centres and CHWs at no cost to patients. Although access to antimicrobials from public facilities is usually given after a clinical assessment and diagnosis as found in our study, this is limited by various health system challenges. Similar to our findings, other studies have also shown that challenges such as stock-out of essential medicines, long distances between community dwellings and health facilities, high cost of transportation to facilities, and long waiting times restrict access to antimicrobials in the public health sector [36, 37]. The role of CHWs in dispensing antimicrobials addresses some of these challenges since they work closer to the communities. However, CHWs only manage three diseases—pneumonia, malaria and diarrhoea—and only among children under 5 years of age as part of integrated community case management of childhood illnesses. In addition, CHWs in Uganda also face regular stock-out of antimicrobials [38, 39]. These challenges are not specific to antimicrobials, but reflect the general constraints on primary health care services in Uganda and other LMICs [40]. Addressing these health system challenges by key stakeholders such as the Ministry of Health would increase access to antimicrobials at public health facilities.

Our study established that the challenges in accessing antimicrobials from the public health sector led community members to resort to private health facilities including drug shops, clinics and pharmacies. Private facilities are crucial for access to essential medicines in LMICs including Uganda [41]. However, as our study revealed, access to antimicrobials from private facilities presents other concerns. The most prominent disadvantage is the out-of-pocket cost to individuals associated with the private sector [7] which implies that although antimicrobials may be available, community members may face financial constraints accessing them. This may in turn result in an insufficient course of antibiotic being afforded. In addition, unlike public facilities which only provide antimicrobials after medical examination and diagnosis, the study revealed that private facilities provide antimicrobials without necessarily doing appropriate clinical assessment nor having a prescription. This practice is common in LMICs and can be attributed to poor regulation of access to antimicrobials [7]. For example, extensive use of antimicrobials without prescription has been reported in other LMICs such as Bangladesh and Sudan [32] and Ethiopia [31, 35]. A systematic review of antimicrobial prescription in the WHO African region showed that private facilities were more likely to provide antimicrobials without necessary clinical assessment in order to retain their customer-base [42]. Another concern with unregulated private facilities is that some antimicrobials accessed may be substandard and counterfeit [7, 43]. Therefore, there is a crucial need for a robust inspection and enforcement structure to regulate access to antimicrobials in the private sector and ensure adherence to existing national guidelines and regulations on antimicrobial prescription [35].

The challenge of accessing antimicrobials for animals without the necessary diagnosis and prescription was evident with farmers heavily reliant on private providers. Indeed, the farmers involved in our study mainly accessed antimicrobials through private providers including mobile veterinary practitioners, as well as veterinary and human drug shops. The use of mobile veterinary practitioners, also commonly known as mobile clinics or units in LMICs, is an innovative solution to promote access to animal health service delivery, especially in rural communities [44]. Although our study revealed gaps in competence and practice of the mobile veterinary practitioners, past studies involving qualified veterinarians showed the provision of quality services. For instance, a recent pilot of mobile veterinary clinics in Kenya used trained agrovets overseen by government veterinarians [45]. The study in Kenya highlights the need to regulate private providers of antimicrobials for animal health which should be fully embraced in Uganda. The lack of regulations and minimal implementation of existing guidelines regarding accessing antimicrobials for animals has implications for human health through contributing to AMR [4]. Therefore, the veterinary capacity of public institutions needs to be strengthened so as to better regulate the use of antimicrobials in animal husbandry. Uganda, like other LMICs such as Kenya and Nigeria, still lacks a strong veterinary health system due to a shortage of veterinary professionals, inadequate funds, and the lack of a clear policy framework [45, 46]. The predominant reliance on private actors will continue to grow unless gaps in the public sector are addressed. Therefore, there is an urgent need for more active government involvement in veterinary service provision in Uganda including regulation of private practice.

Despite the challenges faced in accessing antimicrobials from public and private facilities, our study revealed inappropriate use in humans and animals such as self-medication, sharing of antimicrobials among family members, incomplete dosage consumption, administering human antibiotics in animals, and overuse of antimicrobials in humans and animals. Inappropriate use of antimicrobials is common in LMICs and is among the most critical drivers of AMR globally [31, 32]. The development of AMR from misuse of antimicrobials can occur through direct consumption or indirectly through the presence of residual antimicrobials in animal products or in the environment. Inappropriate use of antimicrobials has led researchers to call for a balance between access and resistance [1], as unregulated access drives indiscriminate behaviour and practices at individual level [35, 42]. Although unregulated access is a crucial enabler of inappropriate consumption of antimicrobials [31], other concerns such as cost, literacy level, geographical location, and low awareness of AMR are also important [7, 31, 40]. For instance, in a study conducted among pastoralists in Kasese region of Uganda, 78% of the respondents did not know about AMR [47]. Therefore, in addition to implementing robust regulatory and surveillance systems, there is a crucial need for increased awareness of appropriate antimicrobial use at community level as stipulated in the Uganda AMR NAP [9] and WHO recommendations [35].

The participants in our study commonly exhibited poor practices in disposing of antimicrobials through burning, and dumping with general waste including in rubbish pits. Poor disposal of antimicrobials is recognized as a global challenge because antimicrobial residues in environmental locations such as soil and water can drive AMR development [23]. While progress is being made in high-income countries by establishing national drug disposal systems such as drug return for safe disposal, evidence on how this can be implemented and sustained in LMICs is still sparse [23, 24, 48]. In addition, poor drug disposal practices are associated with low awareness of proper disposal mechanisms [23, 26]. Although a study in Ethiopia showed high awareness of the environmental impacts of poor antimicrobial disposal, there existed a gap between knowledge and practice due to the contextual factor of lack of a suitable drug disposal system at community level [23]. For effective design of antimicrobial waste management systems, Environmental Health professionals need to be involved. Once such systems are in place, key stakeholders including human and animal health professionals as well as CHWs need to take active roles in educating the community on proper disposal of antimicrobials. Involving other health professions in establishing systems for disposal of antimicrobials through multidisciplinary collaboration [34, 49] will help to address the growing challenge of AMR.

This study was conducted in peri-urban and rural settings in Wakiso district, hence the findings may not be generalizable to the entire district or country. Wakiso district being close to Kampala, the capital city, facilitates access to antimicrobials from private facilities particularly for animals. Districts which are further from the capital may not have similar access, thus the situation regarding access and use of antimicrobials in such communities may differ from those presented in this study. Use of qualitative methods in our study enabled exploration of key issues concerning access and use of antimicrobials in the community including among humans and animals. Another strength of the study was the involvement of a wide range of participants including community members, farmers and key stakeholders at national and sub-national levels which enriched the findings. The assessment of antimicrobial disposal routes in the community is another strength of the study as this has not been fully investigated in Uganda and beyond.

## Conclusion

Antimicrobials for humans were accessed from the public sector including health centres, and private health facilities such as drug shops and clinics. For animals, antimicrobials were predominantly obtained from veterinary practitioners and drug shops (for both veterinary and humans). Inappropriate use of antimicrobials in both humans and animals was reported such as sharing antibiotics among household members, and giving human-prescribed antimicrobials to food-producing animals as growth promoters. Antimicrobials were most commonly disposed of by households with general waste including dumping in rubbish pits. There is a need to increase awareness among the population on proper access, use and disposal of antimicrobials in both humans and animals to help prevent the further development of AMR in communities. In addition, development of a drug disposal system for use in communities is important to facilitate improved waste management of antimicrobials.

## Data Availability

Data from the study are available from the corresponding author on reasonable request.

## Abbreviations

AMR: Antimicrobial resistance
CHW: Community health worker
FGD: Focus group discussion
KII: Key informant interview
LMIC: Low- and middle-income country
NAP: National Action Plan
WHO: World Health Organization
